# Discourse on Dr. Drake

**Published:** 1853-02

**Authors:** 


### DISCOURSE ON DR. DRAKE.

We give up our present number almost entirely to the eloquent discourse of Dr. Gross on Dr. Drake. Apart from its intrinsic interest, we believe the eminent abilities of the subject of the memoir, his long service in the profession, his reputation as an author and teacher, and his connection with this Journal for many years as Senior Editor, will induce a very general desire to read such a narrative of the principal incidents of his eventful life. We are sorry that we could not give it all in this number; but our March issue will be pushed through the press with as little delay as possible, so that the conclusion will soon follow.

